# Environmental Diagnosis through a Flow Cytometric Approach

**DOI:** 10.3390/ijms252011069

**Published:** 2024-10-15

**Authors:** Giovanna Panza, Fabrizio Frontalini, Caterina Ciacci, Giuseppe Protano, Mariele Montanari, Daniele Lopez, Francesco Nannoni, Stefano Papa, Claudio Ortolani, Federica Rebecchi, Vieri Fusi, Riccardo Santolini, Barbara Canonico

**Affiliations:** 1 Department of Biomolecular Sciences (DISB), University of Urbino Carlo Bo, 61029 Urbino, Italy; g.panza1@campus.uniurb.it (G.P.); caterina.ciacci@uniurb.it (C.C.); mariele.montanari@uniurb.it (M.M.); d.lopez1@campus.uniurb.it (D.L.); stefano.papa@uniurb.it (S.P.); barbara.canonico@uniurb.it (B.C.); 2 Department of Pure and Applied Sciences (DiSPeA), University of Urbino Carlo Bo, 61029 Urbino, Italy; fabrizio.frontalini@uniurb.it (F.F.); f.rebecchi@campus.uniurb.it (F.R.); vieri.fusi@uniurb.it (V.F.); 3 Department of Physical, Earth and Environmental Sciences (DSFTA), University of Siena, 53100 Siena, Italy; giuseppe.protano@unisi.it (G.P.); nannoni@unisi.it (F.N.); 4 Department of Humanistic Studies (DISTUM), University of Urbino Carlo Bo, 61029 Urbino, Italy; riccardo.santolini@uniurb.it

**Keywords:** marine, terrestrial, bioindicator, biomarker, hepatopancreas, haemolymph

## Abstract

In an era when ecological and environmental needs and responsibilities apply pressure on the world’s countries and sustainability takes centre stage, ecologic/environmental (E/E) laboratories stand as beacons of scientific inquiry, innovating, optimising, and applying various tests for a better knowledge of our natural resources and the quality status of ecosystems. The purpose of this review is to provide an overview of the use of flow cytometry (FC) as a tool for assessing environmental quality, mainly using living organisms and their biological changes as bioindicators. Cytometric approaches applied to both marine and terrestrial ecosystems ensure the detection of biochemical and functional status of the cells composing either an organ thereof or the organism itself. In addition to cytometric evaluations of the biotic matrix, a brief overview of the techniques for the environmental assessment of biotic and abiotic matrices using mass spectrometry is given. The technique involving the continuous monitoring of the chemical and physical parameters of water, sediment, and soil is basically incapable of detecting any additive and synergetic effects of toxicants on living organisms. Therefore, techniques employing bioindicators provide valuable information for environmental diagnosis, and several studies have demonstrated the strong relationship between specific environmental data and cell/organ behaviour.

## 1. Introduction

The current review fits well in the Special Issue “The Trends and Prospects of Flow Cytometry in Cell and Molecular Biology”, since it includes several of the concepts and keywords indicated, in particular the following: (1) biochemistry, (2) cell function, (3) new applications, and (4) new methods. The highlights of this review article include the main role of flow cytometry (FC) in the environmental field, through the setting up and optimisation of FC protocols, which are able to test biochemical (Reactive Oxygen Species or ROS content, phosphatidylserine flip-flop, and glutathione content) and functional (proliferation, mitochondrial membrane potential, and lysosomal network) parameters. In their general application (e.g., in an environmental bioindication context), these parameters could synergistically work to construct a useful “bioindication index”. In this regard, we want to emphasise the differences between the definition of bioindicators and that of biomarkers. Bioindicators are organisms used to assess the health of ecosystems, while biomarkers are biological changes in organisms that indicate environmental stress or pollution. Using the appropriate bibliography, we can recognise which biomarkers are used on bioindicators and which can be functional for environmental monitoring through FC. In the last part of the current review, a bibliometric analysis was performed to identify the main patterns, trends, and perspectives on cytometric approaches applied to marine and terrestrial ecosystems.

### 1.1. Bioindicators: What They Are and Why to Use Them

**Figure 1:**
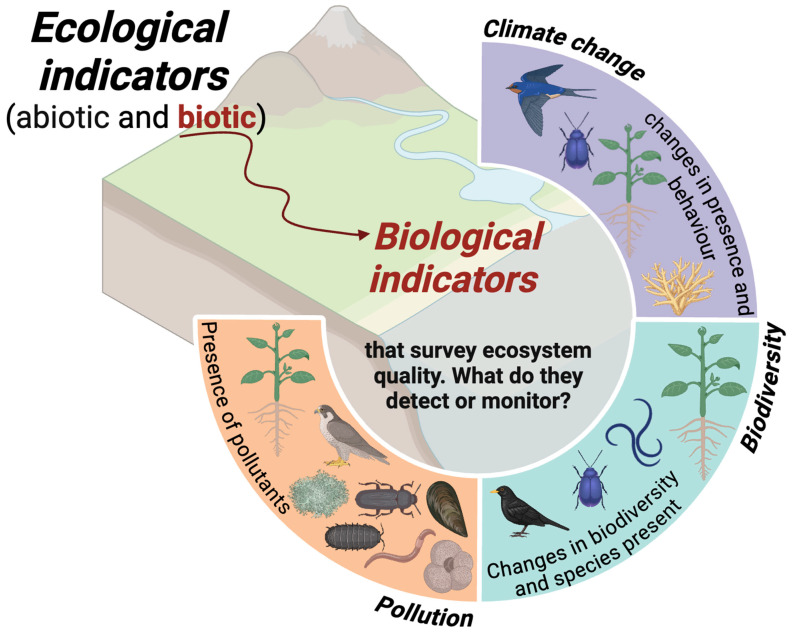
Schematic diagram showing different biological indicators to assess pollution (plants and animals—earthworms, bivalve molluscs, foraminifera, insects, crustacea, lichens, raptor, etc.); biodiversity (animals, plants, and microbial communities); and climate change (plants and animals—migratory birds, insects, and coral). Created in biorender.com, accessed on 1 June 2024.

The use of living organisms to assess environmental stress and pollution levels has considerably developed over the years and is today a common practice [1,2,3,4]. Bioindicator has been defined in many ways, but all refer to an organism (or part of it or a community of organisms) that provides valuable information on the environmental quality (or part of it) [5,6]. On the other hand, a biomonitor is an organism (or part of an organism or community of organisms) that contains information on quantitative aspects of environmental quality [5,6]. Both are biotic ecological indicators (Figure 1). Some of these organisms can uptake pollutants from their environment and, for this reason, can be used as indicators of the bioavailability of contaminants [7]. Therefore, bioindicators and biomonitors can be used for the qualitative and quantitative assessments of environmental factors caused by human activities that are altering the ecosystem’s balance [1,5,6,8]. Biomonitoring is defined as the use of organisms/materials to obtain information on ecosystems [9]. The effects of environmental alterations on bioindicators/biomonitors (e.g., plants or animals) may include changes in their physical characteristics (e.g., morphological, histological, or cellular structure), metabolic–biochemical processes (e.g., accumulation rates), or behaviour or population structure (impact: species composition and/or richness, physiological and/or ecological performance, morphology) [5,6,9].

Figure 1 shows examples of ecological indicators, particularly biological (biotic) indicators, and the information they can provide.

The degree of environmental contamination is traditionally measured by analytical techniques on abiotic samples (e.g., soil, sediment, or water samples). This approach has certain advantages, such as the direct interpretation of data, rapidity, and analytical precision; however, from an ecological point of view, this approach is accompanied by relevant problems. In fact, spatio-temporal fluctuations in contaminant emissions can lead to interpretation “bias”, possibly of considerable magnitude. This mainly occurs when intermittent or sporadic emissions are present, whereas biological indicators can record them. Indeed, the continuous monitoring of the chemical and physical parameters of water, sediments, and soils is an approach incapable of detecting any additive and synergetic effects of toxicants on living organisms [10,11].

For these reasons, bioindicators have become increasingly popular and provide valuable insights into the management of environmental resources. However, there is a need to identify new and effective bioindicators to monitor and observe environmental changes [12,13]. Monitoring using biological indicators is not an alternative to chemical assessments of the environment conditions (e.g., heavy metal contents in soil samples). Still, it can provide useful information for identifying possible risk areas [14].

### 1.2. Biomarkers: Different Meanings and Applications

In addition to traditional floristic, faunistic, and biocenotic surveys that typically record non-specific reactions to pollutant exposure at higher organismic levels, several new methods have been introduced in bioindication. These methods include the application of biomarkers that can reveal impact events even before measurable effects appear in the biocenosis and at the population level [15,16].

**Figure 2:**
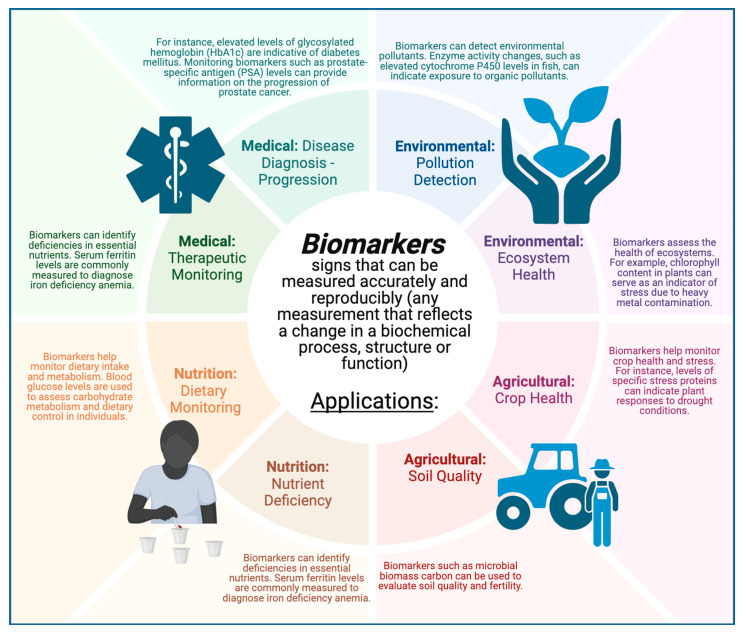
The main fields of application of biomarkers and the information they can provide. Created in biorender.com, accessed on 3 June 2024.

Biomarkers are measurable biological parameters at the sub-organismic level, such as genetic, enzymatic, physiological, or morphological changes, which indicate environmental influences in general and, in some cases, the action of specific pollutants in qualitative and quantitative terms [6,17]. Biomarkers can be biological indicators that can also be used to detect and measure various physiological states or environmental conditions [18,19]. Their meanings and applications can widely vary depending on the context in which they are used. Today, they find applications in medical (e.g., disease diagnosis, disease progression, and therapeutic monitoring), environmental (e.g., pollution detection and ecosystem health), agricultural (e.g., crop health and soil quality), and nutritional (e.g., nutrient deficiency and dietary monitoring) fields. Biomarkers are versatile tools that provide significant insights by reflecting physiological, pathological, and environmental states. Their applications extend from clinical diagnostics and therapeutic monitoring to environmental assessment and agricultural management, highlighting their critical role in advancing scientific understanding and practical applications. In Figure 2, the main fields of application for biomarkers are reported.

Biomarkers can reveal the presence of pollutants in the environment by showing physiological changes in organisms, like increased levels of specific enzymes in fish exposed to contaminants (pollution detection). They can help assess the overall health of ecosystems by measuring indicators like chlorophyll concentration in plants to monitor stress due to pollution (ecosystem health). It is essential to consider the variables that are inevitably involved (e.g., seasonality, temperature, pH, humidity, etc.) when using biomarkers to assess effects due to contaminants, so that the contribution of these variables can be excluded to effectively evaluate the impact of the pollutant [20].

### 1.3. The Breakthrough of FC in the Immunology of Invertebrates

**Figure 3:**
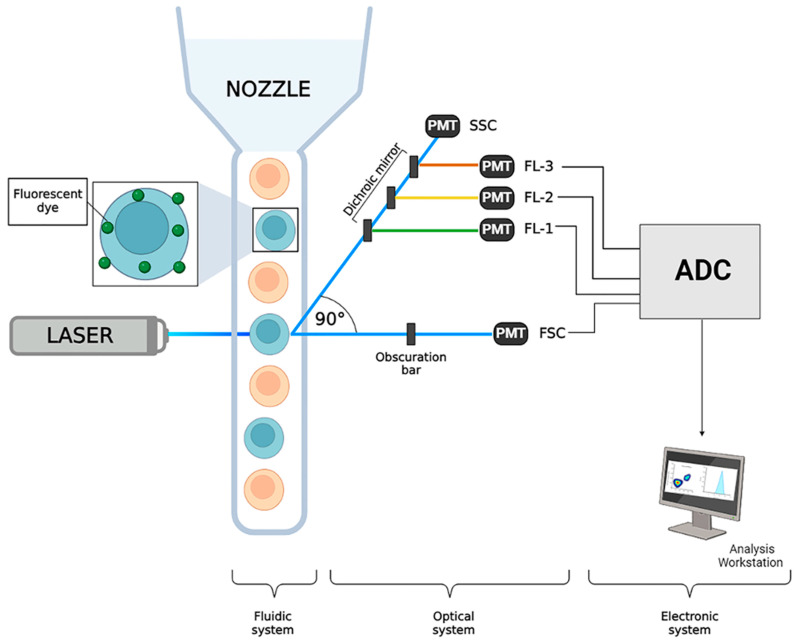
Flow cytometer’s fluidic, optical, and electronic systems depicted in a schematic diagram. Photomultiplier tubes (PMTs), Fluorescent channel (FL), Side Scatter (SSC), Forward Scatter (FSC), and analogue-to-digital converters (ADCs). Created in biorender.com, accessed on 5 June 2024.

Cooper et al. [21], in the book “Advances in Comparative and Environmental Physiology—Invertebrate Immune Responses: Cells and Molecular Products”, stated that flow cytometry (FC), though able to analyse extremely high numbers of cells in a few minutes, is not commonly used in the immune system of the invertebrates, as it is in mammals. Our review underlines the great contribution and competitiveness of FC in this field, due to representing a methodology that enables fast, quantitative, and multi-parametric analyses. This single-cell technology enables the analysis of thousands of cells in a few seconds. It represents, therefore, a rapid technique that can be applied to both morphological and functional studies of cells in suspension [22,23]. The FC analytical technique can analyse particles, known as events, virtually as singlets through a fluid (hydrodynamic focusing); this creates a single-cell stream that passes in front of a laser. Collecting and digitising the signals produced by this interaction enables researchers to obtain quantitative and qualitative information on the investigated parameters of the events. FC can be used to evaluate particles of different kinds and sizes. Visible light scatter is assessed in two directions: forward (forward scatter or FSC), which reveals the relative size of the cell, and at 90° (side scatter or SSC), which denotes the cell’s internal complexity or granularity. Samples are prepared for fluorescence measurement by staining them with fluorescent dyes (e.g., propidium iodide, DNA) or staining with fluorescent antibodies, which precisely quantify the structural and functional properties of a cell (or particle) (Figure 3).

Flow cytometry is a powerful tool with great applications in immunology, molecular biology, bacteriology, virology, cancer biology, infectious disease monitoring, and bioindication studies.

Fluorescent probes employed in FC analysis might be membrane-bound, cytoplasmic, or linked (and labelling) to nuclear material: they can be collected and attributed to a specific parameter (Figure 3). To identify specific receptors on the plasma membrane, as well as intracellular antigens or the quantity of a particular molecule within a cell, it is common to use monoclonal or polyclonal antibodies that are directly conjugated to fluorescent dyes [24]. In fact, the first FC studies in invertebrates were applied to the analysis of epitopes/molecules present in cells from the mollusc *Planorbarius corneus*, which were able to cross-react with human molecules [25].

The FSC and SSC parameters can be used to distinguish different subpopulations in the haemolymph of both terrestrial (*Armadillidium vulgare*) and marine invertebrates (*Mytilus galloprovincialis*). When combined, FSC and SSC allow for the identification of heterogeneous cellular samples within different populations [22,26]. Different types of haemocytes have different functions [27]. Hence, it is important to identify cell subpopulations to understand the immune functions of each cell type [28] across various invertebrate taxonomic classes such as molluscs, echinoderms, insects, and crustaceans. Besides immunocytes/haemocytes and, commonly, immune cells [21,29], other cell types have also been considered to gather information on the environment in which animals live, such as the following: midgut tissues in beetles [30], in *Locusta migratoria* [31], alimentary canals in honeybee workers [32], and hepatopancreas in the terrestrial isopods *A. vulgare* [14]. The need for efficient tissue disaggregation procedures is increasingly pressing since, as cited, not only is haemolymph (and haemocytes) collected and analysed but also solid tissues, which should be disaggregated with the least possible impact and minimal manipulation [33,34].

## 2. Biochemical and Functional Tests to Study Ecosystem Health in a Laboratory Setting

**Figure 4:**
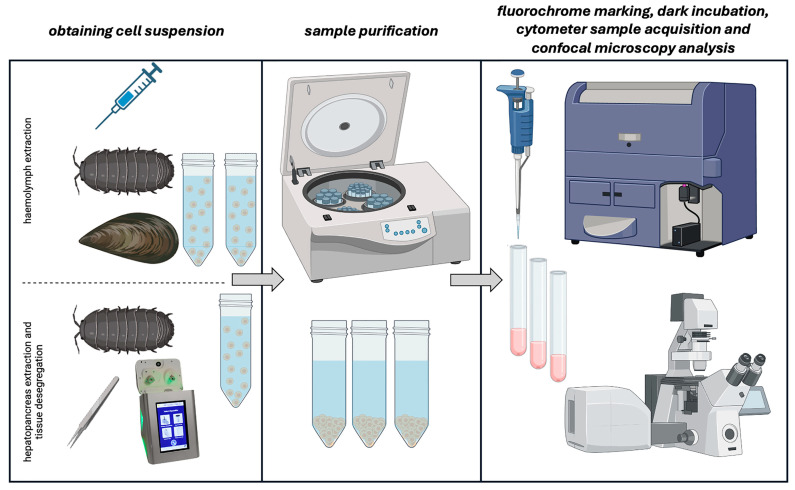
Schematic diagram of protocol steps for analysing haemolymph (isopods and mussels) and cells from the hepatopancreas tissue after disaggregation (isopods) using flow cytometry and confocal microscopy. Created in biorender.com, accessed on 7 June 2024.

The physiological conditions of an organism exposed to environmental stressors can be assessed using numerous biochemical and molecular indicators. The FC applications in various fields (e.g., marine biology, molecular biology, and immunology) have emerged due to recent advances in instrumentation, software, and fluorochrome chemistry [28] (Figure 4).

In contrast to vertebrates, bivalves rely mainly on their innate immune system for their defence mechanisms. Bivalves have a non-specific innate defence system comprising cellular components like haemocytes, epithelial cells, and soluble components released by haemocytes in the haemolymph [28]. Most immunological studies in bivalves have focused on haemocytes, a component of the haemolymph. FC has made it possible to characterise bivalve haemocytes more comprehensively, including different quantitative parameters (e.g., cell viability, total cell count, subpopulation classification, oxidative stress, apoptosis, and phagocytosis) [35,36]. In the following chapters, the expertise of our group, particularly in the field of the marine environment, will be reported. Additionally, in Section 2.2 we will focus on the following: (a) recent advances in newly optimised tests to detect micro- and nanoplastics discharged into wastewater from a different source, representing a direct FC measurement of the specific pollutants; (b) recent advances reached in the immunology of marine mammals; and (c) the viability and function of benthic foraminifera, using confocal microscopy analysis (CMA) for both.

### 2.1. Bioindicators and Biomarkers: Flow Cytometry Works Well

Marine invertebrates can be considered a significant target group for evaluating the effects of different environmental contaminants. Bivalve molluscs, which are filter feeder organisms, are good bioindicators due to their worldwide distribution (from freshwater to marine ecosystems), sedentary behaviour, and the low costs of sampling them. In the presence of emerging contamination (e.g., nanoparticles), bivalves may play an important role in the uptake, biotransformation, and transfer of these compounds through food webs [37,38,39,40,41,42]. The contaminants act as environmental stressors and the physiological or “pathologic” conditions of the exposed organism can be evaluated using biochemical and molecular indicators. Such indicators can be translated into a “cytometric setting” as follows, at least based on our experience: (i) the loss of cell viability (PI, 7-AAD, Annexin tests) [43,44]; (ii) oxidative stress (MitoSOX, DCF, GSH tests) [45,46]; (iii) mitochondrial toxicity (TMRE, MitoTracker, JC-1 tests) [47,48]; (iv) lysosome network impairment (LysoTracker, LysoSensor tests) [49,50,51]; (v) cell cycle phases alteration (DNA probes on fixed cells) [52]. These analytical techniques provide important information for environmental diagnosis and have been related in many studies to the responses of organisms [22,53].

Here, we provide the readers with the following list of applications (and brief protocols) from our more recent studies, as well as from more innovative research in environmental fields that have successfully utilised the FC approach:

1. In the paper of Canesi et al. [54], the effects of 50 nm amino-modified polystyrene nanoparticles (PS-NH_2_) were investigated in the marine bivalve *M. galloprovincialis* haemocytes. FC was employed to obtain the haemocyte absolute counts and dead, apoptotic, and necrotic cells simultaneouslyand in real-time, using Annexin V-FITC and propidium iodide (PI) tests.
2. In the same study [54], the investigation of the apoptotic process was analysed more in depth, allowing for the detection of the effects of PS-NH2 on the mitochondrial membrane potential (MMP, Δψm), evaluated by the fluorescent dye tetramethylrhodamine, ethyl ester perchlorate (TMRE). TMRE is a quantitative marker used to measure the maintenance of the MMP. It accumulates in the mitochondrial matrix based on the Nernst equation. TMRE specifically stains mitochondria and is not present in cells when the Δψm collapses, which is an early stage in apoptotic processes [54]. Indeed, in addition to the apoptotic process, mitochondria also provide complex information from the environment and intracellular milieu, including the presence of reactive oxygen species (ROS) and toxic substances [55].
3. Mitochondria can be further analysed using FC, specifically concerning the composition of their inner membrane, successfully employing the cardiolipin (CL) sensitive probe, 10-nonyl-acridine orange (NAO), which is able to sense CL peroxidation [54].
4. Subsequently, Auguste et al. examined the impact of repeated exposure to PS-NH_2_ on the immune responses of *M. galloprovincialis* [35]. The study involved an initial exposure of 24 h, followed by a rest period (with a 72 h duration), and then a second exposure of another 24 h. FC was used to determine the total haemocytes count (THC) and to characterise various cell types in mussel haemolymph from both control and PS-NH_2_-exposed mussels under different experimental conditions. It is important to note that the FC methodology feature enables the use of specific gates to distinguish the different cell subpopulations, as well as to exclude spermatozoa, cell debris, and aggregates from analyses.
5. Finally, our FC group addressed oxidative stress at the single cell level, reporting data on C-DCF (cytosolic- H2DCFDA), MitoSOX (Mitochondrial Superoxide Indicators), and GSH (Intracellular Glutathione) probe labelling [36] on each event belonging to each of the heterogeneous subpopulations of the samples. The setup protocols were optimised, starting from yet-to-be-applied protocols for humans, and are efficient at collecting precise oxidative stress parameters and monitoring the possible peak in oxyradical production at mitochondrial (MitoSOX) and cytosolic (C-DCF) levels.
6. FC was also employed recently [14] to evaluate the heavy metal content, using stains such as Leadmium Green [56], in terrestrial isopods. These aspects will be discussed more in depth in Section 2.2 (Terrestrial ecosystems: recent advances). Furthermore, FC is an essential tool for the detection of the efficacy of yet uncommercialised fluorophores: the Fly probe, developed by a research group from Urbino [57,58], for example, is helpful when tracing divalent metals (i.e., copper Cu^2+^).

### 2.2. Marine Ecosystems: Recent Advances

FC in marine ecosystems, particularly for organisms such as mussels, is used to analyse the cells’ size, complexity, and biochemical markers.

In detail, FC monitors mussel haemocytes (immune cells). The technique can assess immune function by measuring changes in the size, granularity, and internal structures of cells, often using staining to detect oxidative stress or other immune responses. In contrast, for foraminifera, fluorescent probes (using a CMA approach) help to analyse the calcified shells and cytoplasm of these microorganisms, often by examining cell size, pigment content (e.g., chlorophyll in symbiotic algae), or acidic vacuoles.

Several factors cause changes in the cytometric signal; for example, changes in temperature, salinity, or pH can affect mussels, inducing cell size alterations, organelle morphology, functional changes, or deep variation in self-fluorescence. Pollutants can alter metabolic or immune responses, which can then be rapidly recorded and quantified using FC and CMA.

Therefore, FC provides valuable real-time data for these marine organisms affected (and not affected) by environmental stressors and pollutants. Data are collected on viable “single” cells in multicolour, and they then require careful interpretation.

(a) FC is currently employed not only to evaluate cells and tissues from bioindicators but also directly on aquatic environmental matrices, particularly marine ones. In recent years, the utilisation of FC for quantitative microplastic analysis has gained importance and visibility [59,60,61]. By improving FC-based protocols, Li [61] offered key insights for assessing microplastic and nanoplastic toxicity. Currently, the most used methods for microplastic counting are Raman spectroscopy and microscopy [62]. Nevertheless, combining multistage filtration, Nile Red (NR) staining, and flow cytometry has established a quantitative analysis method for microplastics and nanoplastics [61].

(b) Marine mammals, located at the apex of the aquatic food chain, are distinguished by their extended lifespans and can become the final recipients of contaminants within marine ecosystems. These effects can result from a variety of hazards, including pollution [63]. Exposure to various pollutants, such as polychlorinated biphenyls (PCBs), pesticides, or heavy metals, can cause animal disorders. Several in vitro investigations conducted with different marine mammal species have shown a clear association between exposure to xenobiotics and changes in immune function. Filipo-Benavent and coworkers [63] determined the phagocytic activity of monocytes and granulocytes of bottlenose dolphins, beluga whales, Patagonian sea lions, walruses, and harbour seal using FC. They provided the physiologic range to which to relate the alterations induced by possible environmental disturbances.

(c) Benthic foraminifera, single-celled organisms, have been increasingly applied as bioindicators of environmental stress (e.g., pollution and confinement) in coastal and marginal marine ecosystems [64]. These organisms play a crucial role in global biogeochemical cycles. They are sensitive to environmental changes, such as the temporal variation of physico-chemical parameters, sediment composition, pollutants, and the availability of organic matter and oxygen, among others [65]. Because of the complex interplay of these parameters, disentangling the natural vs. human-induced stresses is difficult. Quantitative responses on benthic foraminiferal viability and physiological health can be alternatively achieved using laboratory experiments. In this context, a wide array of fluorescent and fluorogenic probes has been applied to observe cellular ultrastructure and the cells’ metabolic processes, as well as to infer the physiological state of foraminifera [66]. For instance, acridine orange, a pH-sensitive dye, has been used to detect and quantify acidic vesicular organelles in a foraminiferal species when exposed to Hg and titanium dioxide nanoparticles [67,68]. NR, a phenoxazine dye, has been tested to localise and quantify neutral and polar lipids on benthic foraminiferal cells treated with Hg, Cd, and several nanoparticles (i.e., titanium dioxide, polystyrene, and silicon dioxide) [67,69,70]. CellROX has been applied to detect ROS within foraminiferal cells incubated with titanium dioxide, polystyrene, and silicon dioxide nanoparticles [68,69]. Additional fluorescent and fluorogenic probes (e.g., Hoechst 33342, MitoTracker, SiR-actin) have been used to observe cellular processes and ultrastructure [66] (for a review). Most of these probes have found applications in FC as well.

### 2.3. Terrestrial Ecosystems: Recent Advances

FC in terrestrial ecosystems, is used to study immune cells (haemocytes), cell viability (correlated to organ health), and stress response in species such as Isopoda.

All these factors can be monitored using FC, particularly in in vitro models (by comparison with the control condition) but also under in vivo conditions, especially when collecting bioindicator organisms from different polluted sites, in which FC can efficiently detect changes induced by environmental factors such as pollutants, infections, or other stressors.

Furthermore, one of the most significant advantages of FC is its ability to rapidly process large numbers of samples with minimal sample preparations. Traditional techniques, like atomic absorption spectrometry (AAS) and inductively coupled plasma mass spectrometry (ICP-MS), commonly require extensive sample preparation (e.g., digestion and filtration), which adds time to the analysis. Finally, FC can quickly and efficiently associate the lack of intracellular heavy metal content with a limited number of viable cells that are incapable of accumulation processes and even of responding with their antioxidant machinery. This is an important step that, if missed, could erroneously suggest a favourable state of environmental health due to the absence of accumulated heavy metals.

The high bioindication capacity of Isopoda (Crustacea, Oniscidea) has been reported in many studies, as well as its ability to accumulate contaminants [71,72]. Soil ecotoxicology uses isopods as relevant models in laboratory toxicity tests and field monitoring [72,73]. Isopods are terrestrial invertebrates that offer insights into the levels of soil contamination [71,72,74]. In particular, the hepatopancreas has been identified as the primary tissue for contaminant accumulation. For example, it accumulates heavy metals from various sources such as agriculture and industry [14,72,75,76]. The hepatopancreas contains two cell types, the Big (B cells) and the Small (S cells) cells, which have different excretion behaviours [14,72,75]. The S cells accumulate metals, while the B cells are renewed frequently, playing the main role in excretion [72,77].

In Panza et al. [14], the cell functions and viability of the hepatopancreatic cells (S and B cells) of isopods from sites with different degrees of ecological disturbance were analysed by FC to detect differences in stress parameters, finally verifying if these changes/alterations corresponded to the environmental stress condition to which they were subjected. Several markers for cell functions (e.g., viability, mitochondria and lysosomal network, oxidative stress, and heavy metal content) were employed on the cell suspension. They highlighted a low level of cellular damage in apparently uncontaminated areas, an intermediate level of cellular damage in variously urbanised areas, and high levels of damage in urban sites (i.e., industrial areas). Significant differences in cell functional parameters were found, highlighting the efficiency of analysing isopod hepatopancreatic cells using FC. Indeed, they revealed higher percentages of dead cells (both S and B cells) in individuals from highly polluted sites, compared to those from apparently unpolluted areas.

In Manti et al. [53], a component analysis was conducted to understand the relationships between the S and B cells, as measured by FC and chemical elements. The data supported a relationship between heavy metals (bound into leachate) and S cells, which were more efficient than B cells at metal storage. The authors employed FC to provide detailed information on the cellular status and effects of metal bioavailability, highlighting FC as an invaluable tool for environmental scientists as well. As FC continues to evolve, its application in heavy metal detection would likely expand, currently complementing or, in the future, even replacing traditional methods.

#### Relationship between Soil Chemistry and Metal Content in Isopods

An innovative and interesting approach in environmental and ecotoxicological research is the assessment of the biological effects of heavy metals on cells of terrestrial invertebrates such as isopods by combining mass spectrometric and FC analyses.

In this field, Manti et al. [53] analysed, using ICP-MS, the concentrations of heavy metals (As, Cd, Cr, Cu, Ni, Pb, V, Sb, and Zn) in specimens of *A. vulgare* isopod exposed to the leachate of a municipal solid waste landfill, and determined, using FC, the physical characteristics and functional parameters of different-sized hepatopancreatic cells (S and B cells). They identified a relationship between heavy metal concentrations in isopod tissues and the biochemical and functional status of hepatopancreatic S cells. The use of FC for defining the biological effects of heavy metals on isopod cells arose from the fact that these terrestrial invertebrates (e.g., *A. vulgare*) can uptake heavy metals and accumulate them in their tissues [78,79].

Terrestrial isopods are detritivorous organisms living close to soil and litter in the upper soil profile. These animals may be exposed to heavy metals, as their surface microhabitat and food source may be enriched in these toxic elements through several human activities (e.g., vehicular traffic, agricultural practices, etc.). Moreover, terrestrial isopods are sensitive to environmental changes and alterations due to the contribution of heavy elements. Accordingly, they are considered suitable bioindicators of environmental quality and are used in biomonitoring research to identify possible risks for ecosystems caused by soil contamination by heavy metals [72,80,81,82,83].

Several field and laboratory studies have been carried out to investigate the uptake, accumulation, excretion, and regulation mechanisms of heavy metals by terrestrial isopods [80,84,85]. Terrestrial isopods living on and in the soil may uptake chemicals, including heavy metals, through dermal and intestinal exposure routes. The dermal uptake of heavy metals mainly occurs through the exposure of isopods to soil solution (the liquid phase surrounding the inorganic and organic soil particles). The concentration of heavy metals in a soil solution is ruled by several physical, chemical, and biological factors, among which are reactions and processes both in the soil solution and between this liquid phase and the solid components of the soil. These reactions and processes (e.g., physical and chemical adsorption, precipitation, solubilization, etc.) are controlled by the soil’s physico-chemical properties and composition (e.g., pH, cation exchange capacity, content of organic matter, etc.) and regulate the distribution/partitioning of heavy metals in soil fractions, so-called chemical fractionation [86]. Heavy metal accumulation in a soil solution which becomes available for dermal uptake originates from the following soil fractions: (i) a water-soluble fraction in which heavy metals are in water-soluble phases; (ii) an exchangeable fraction in which heavy metals are adsorbed via ionic exchange on the surface of solid constituents (e.g., clay minerals and organic compounds); and (iii) an acid-soluble fraction in which heavy metals are associated by precipitation and/or co-precipitation with acid-soluble solid constituents, such as carbonates. These water-soluble, exchangeable, and acid-soluble fractions, which together constitute the extractable fraction, represent the most mobile, active, and accessible accumulation of heavy metals in soil, also called the “effective available pool”. The uptake of heavy metals in the isopod intestine mainly occurs through the digestion of ingested soil organic matter (e.g., humic and non-humic substances) that releases heavy metals adsorbed by ionic exchange (exchangeable fraction) and complexation (oxidable fraction) on the surface of the soil’s organic compounds. This fraction of heavy metals constitutes the bioaccessible pool.

Chemical and biological methods have been used to evaluate the availability of heavy metals for soil invertebrates such as isopods. Chemical methods mainly consist of extraction procedures that enable us to define the heavy metal distribution in soil fractions contributing to the effective available and bioaccessible pools for soil invertebrates; but they do not consider the biological factors responsible for uptake. Biological methods assess the availability and bioaccessibility of heavy metals in soil by measuring their accumulation in terrestrial invertebrate cells [87].

## 3. Flow Cytometry in Environmental Diagnosis: Final Considerations

**Figure 5:**
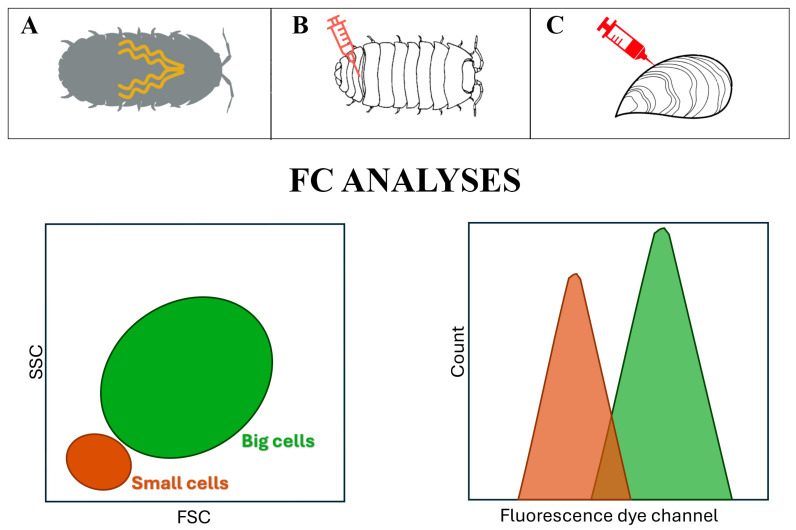
Representative dot plots of FSC vs. SSC and fluorescence histograms for the quantitative evaluation of specific dye MFIs (mean fluorescence intensities) in cells obtained from the following: (**A**), hepatopancreas of *Armadillidium vulgare*; (**B**) haemolymph of *A. vulgare*, and (**C**) haemolymph of *Mytilus galloprovincialis*.

This review reveals and summarises the efficient, functional, and innovative aspects of using FC in the environmental field (Figure 5).

Since its development, FC has proved to be a technique with a transversal nature; in fact, its numerous applications have enabled important discoveries in various scientific fields such as cell biology, immunology, oncology, microbiology, the environmental and food industries, and plant research [23,88,89]. This review examines flow cytometry’s broad environmental applications across multiple sectors, with a focus on bioindicator organisms. Nonetheless, FC applications also include bioremediation, landfills, anaerobic digestion, industrial bioprocesses, water regulation, and soil quality regulation. By conducting an in-depth analysis of the authors’ expertise and of the previous literature, this article sheds light on the potential benefits and challenges of the flow cytometric approach in addressing environmental concerns.

Recent technological advances have led to a comprehensive range of innovative flow cytometers. These compact and user-friendly devices are ideal for conducting routine field sampling in ecology and environmental studies.

A bibliometric analysis was performed to identify the main patterns, trends, and perspectives on cytometric approaches applied to marine and terrestrial ecosystems. This analysis allows us to identify the main themes, trends, gaps in the literature, and opportunities on the topic. To the best of our knowledge, the present scientometric analysis on cytometric approaches to monitor marine and terrestrial ecosystems has not yet been performed.

The search of documents on Scopus as reference database was based on specific keywords, namely “flow cytometry” or “flow cytometric data” or “cytometry” or “cytometric approach” or “FCM” or “FC” or “Facs” and “ecosystem” or “terrestrial ecosystem” or “marine ecosystem” or “invertebrate” or “crustacea” or “isopoda” or “bioindicator” or “one health” or “environmental quality” or “environmental condition”. Since the key theme of this analysis was documents (e.g., articles, conference papers, reviews, and book chapters) focusing on cytometric approaches used to tackle environmental concerns in both marine and terrestrial ecosystems, some keywords, such as “human” and “cancer”, were removed. The analysis did not take into account data from the literature published in 2024.

A bibliometric analysis was carried out to process the available data from the literature with the aid of VOSviewer software (1.6.20 version), following the methodological approach used by Abd Malek and Frontalini [64]. A keywords co-occurrence analysis was performed to generate network maps of the main research topics (i.e., clusters) and temporal trends in keywords.

**Figure 6:**
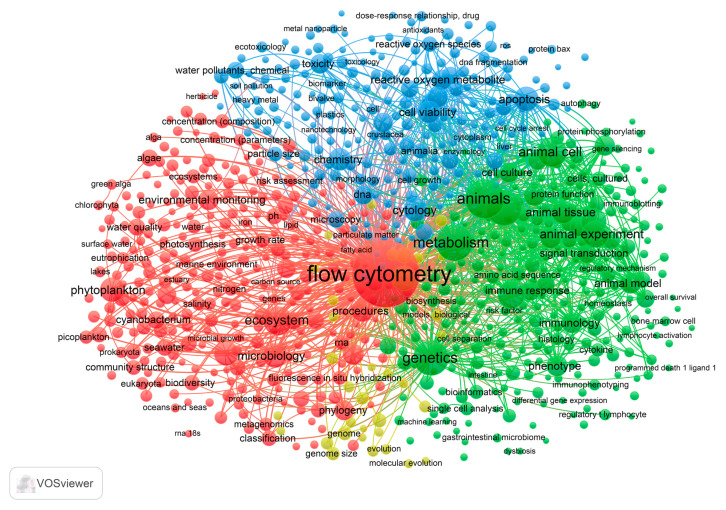
Keyword co-occurrence network map with respect to total link strength.

Overall, 3.074 (from 1973 to 2023) documents were extracted from Scopus, of which the largest proportion (n = 2.751) was scientific articles. Review papers (n = 183), conference papers (n = 68), and book chapters (n = 38) represented a minor proportion. The keywords co-occurrence analysis provided 27.387 results, wherein flow cytometry (n = 2.515), metabolisms (n = 664), animals (n = 784), and genetics (n = 610) represented the top five keywords. Only keywords (n = 3.708) with at least five occurrences were retained. The relevance of keywords, as well as their relationships, were plotted (Figure 6). Based on this, four clusters were identified, which reflected the following: (1) the application of flow cytometry in environmental monitoring (shown in red), for which the frequently co-occurring keywords were flow cytometry, environmental monitoring, ecosystems, microbiology, and phytoplankton; (2) research on flow cytometry-based animal experiments (shown in green) that grouped keywords such as genetics, animals, metabolism, animal cell, animal experiments, and animal tissue; (3) cell-based research (shown in blue) with a high occurrence of keywords such as cytology, apoptosis, toxicity, cell viability, reactive oxygen species, and reactive oxygen metabolites; and (4) genetics-related research (shown in yellow), wherein genome, metagenomics, single cell analysis, and genome size were the prevalent keywords.

**Figure 7:**
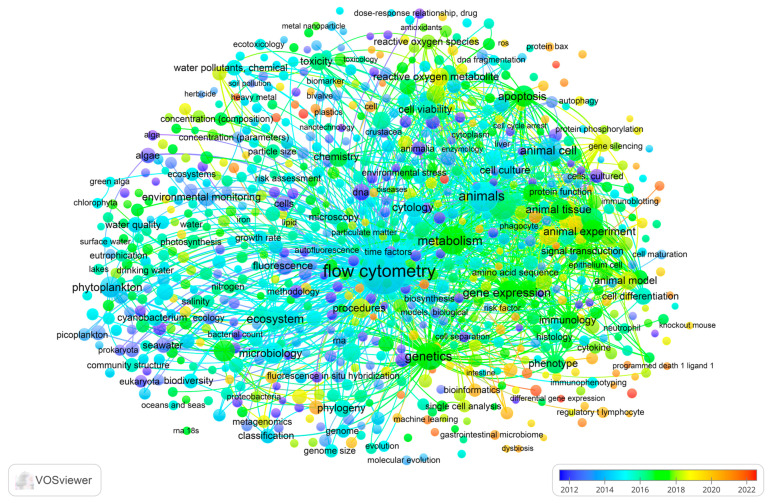
Keyword co-occurrence network map with respect to total link strength with the score of the average publication year of the documents. Overlay network created with flow cytometry and ecological–environmental topics.

The co-occurrence network map of keywords also qualitatively showed the trends of keywords from 2012 to 2022 (Figure 7). It was evident that some keywords reflected a more recent development in the present field. Specifically, plastics, machine learning, differential gene expression, bioinformatics, gastrointestinal microbiome, and immunoblotting (i.e., 2020–2022; red-to-orange colour in Figure 6) are emerging themes that are being explored in biomolecular sciences. It also became evident that the most recent acquisitions in the field (i.e., the green, orange, and red nodes) were related to the application of animal experiments (cluster 2) and cell-based research (cluster 3). On the other hand, the prevalence of the light blue-to-blue colour in cluster 1 reveals that the application of FC in environmental monitoring saw momentum before 2016. This temporal trend clearly identified several pathways, as follows: (i) the transition from the application of FC in the more traditional environmental monitoring to a more cell- and genetic-based one; and (ii) the consideration of emerging pollutants (i.e., plastic).

In agreement with the above, for the 1973–2023 time period, the most recent and recurrent keywords associated with FC are reported in Table 1. It shows that the words “heavy metals” and “immunoblotting” were first associated with FC in 1991. Only in 2016 did the focus shift to “machine learning” and the “gastrointestinal microbiome”. However, in 2003, discussions had already begun to take place on the study of “plastic”, using cytometric analysis, a keyword which then became recurrent in a more environmental perspective from 2008. This reveals how the FC approach, which originated in the early 1990s for counting blood cells, has now a strong potential for new applications and methods in the environmental biomonitoring and genetics fields.

In general, our bibliometric review has provided the basis for a more in-depth study of the application context of FC in environmental diagnosis. Both topics need to be explored and advanced to have a strategic overview of the relationships between flow cytometry and environmental diagnostics, promoting a thorough understanding of innovations, future directions, and potential synergies in research and academia.

## Figures and Tables

**Table 1:** Keywords appearing for the first time associated with flow cytometry.

Keyword	First Time Associated with FC
machine learning	2016
gastrointestinal microbiome	2016
environmental stress	2008
plastic	2003
heavy metal	1994
immunoblotting	1991

## Data Availability

Not applicable.
